# MITE: the Minimum Information about a Tailoring Enzyme database for capturing specialized metabolite biosynthesis

**DOI:** 10.1093/nar/gkaf969

**Published:** 2025-09-26

**Authors:** Adriano Rutz, Daniel Probst, César Aguilar, Daniel Y Akiyama, Fabrizio Alberti, Hannah E Augustijn, Nicole E Avalon, Christine Beemelmanns, Hellen Bertoletti Barbieri, Friederike Biermann, Alan J Bridge, Esteban Charria Girón, Russell Cox, Max Crüsemann, Paul M D’Agostino, Marc Feuermann, Jennifer Gerke, Karina Gutiérrez García, Jonathan E Holme, Ji-Yeon Hwang, Riccardo Iacovelli, Júlio César Jeronimo Barbosa, Navneet Kaur, Martin Klapper, Anna M Köhler, Aleksandra Korenskaia, Noel Kubach, Byung T Lee, Catarina Loureiro, Shrikant Mantri, Simran Narula, David Meijer, Jorge C Navarro-Muñoz, Giang-Son Nguyen, Sunaina Paliyal, Mohit Panghal, Latika Rao, Simon Sieber, Nika Sokolova, Sven T Sowa, Judit Szenei, Barbara R Terlouw, Heiner G Weddeling, Jingwei Yu, Nadine Ziemert, Tilmann Weber, Kai Blin, Justin J J van der Hooft, Marnix H Medema, Mitja M Zdouc

**Affiliations:** Institute for Molecular Systems Biology, ETH Zürich, Otto-Stern-Weg 3, Zürich 8093, Switzerland; Bioinformatics Group, Wageningen University & Research, Droevendaalsesteeg 1, Wageningen 6708PB, The Netherlands; Industrial Genomics Laboratory, Centro de Biotecnología FEMSA, Escuela de Ingeniería y Ciencias, Tecnológico de Monterrey, Av. Eugenio Garza Sada 2501 Sur, Nuevo Leon 64700, Mexico; Department of Organic Chemistry, Institute of Chemistry, University of Campinas (UNICAMP), Rua Monteiro Lobato 270, Campinas, São Paulo 13.083-862, Brazil; School of Life Sciences, University of Warwick, Gibbet Hill Road, Coventry CV4 7AL, United Kingdom; Bioinformatics Group, Wageningen University & Research, Droevendaalsesteeg 1, Wageningen 6708PB, The Netherlands; Institute of Biology, Leiden University, Sylviusweg 72, Leiden 2333BE, The Netherlands; Department of Pharmaceutical Sciences, University of California, 856 Health Sciences Road, Irvine, CA 92697, United States; Center for Marine Biotechnology and Biomedicine, Scripps Institution of Oceanography, University of California San Diego, 9500 Gilman Drive, La Jolla, CA 92093-0212, United States; Helmholtz Institute for Pharmaceutical Research Saarland (HIPS), Helmholtz Centre for Infection Research (HZI), Campus E8.1, Saarbrücken 66123, Germany; Department Anti-infectives from Microbiota, Saarland University, Campus E8.1, Saarbrücken 66123, Germany; Department of Organic Chemistry, Institute of Chemistry, University of Campinas (UNICAMP), Rua Monteiro Lobato 270, Campinas, São Paulo 13.083-862, Brazil; Bioinformatics Group, Wageningen University & Research, Droevendaalsesteeg 1, Wageningen 6708PB, The Netherlands; Institute for Molecular Biosciences, Goethe University Frankfurt, Max-von-Laue-Straße 9, Frankfurt am Main 60438, Germany; LOEWE Center for Translational Biodiversity Genomics (TBG), Senckenberganlage 25, Frankfurt am Main 60325, Germany; SIB Swiss Institute of Bioinformatics, Centre Medical Universitaire, 1 rue Michel Servet, 1211 Geneva 4, Switzerland; Bioinformatics Group, Wageningen University & Research, Droevendaalsesteeg 1, Wageningen 6708PB, The Netherlands; Department of Microbial Drugs, Helmholtz Centre for Infection Research (HZI), Inhoffenstraße 7, Braunschweig 38124, Germany; Institute for Organic Chemistry and BMWZ, Leibniz Universität Hannover, Schneiderberg 38, Hannover 30167, Germany; Institute of Pharmaceutical Biology, University of Bonn, Nussallee 6, Bonn 53115, Germany; Institute for Pharmaceutical Biology, Goethe University Frankfurt, Max-von-Laue-Straße 9, Frankfurt am Main 60438, Germany; Helmholtz Institute for Pharmaceutical Research Saarland (HIPS), Helmholtz Centre for Infection Research (HZI), Campus E8.1, Saarbrücken 66123, Germany; SIB Swiss Institute of Bioinformatics, Centre Medical Universitaire, 1 rue Michel Servet, 1211 Geneva 4, Switzerland; Institute for Organic Chemistry, Leibniz Universität Hannover, Schneiderberg 38, Hannover 30167, Germany; Biosphere Sciences and Engineering Division, Carnegie Institution for Science, 3520 San Martin Dr, Baltimore, MD 21218, United States; Department of Ecology and Evolutionary Biology, University of Arizona, 1041 E. Lowell St., Tucson, AZ 85721, United States; Department of Biotechnology and Nanomedicine, SINTEF Industry, P.O. Box 4760 Torgard, Trondheim N-7465, Norway; Molecular Targets Program, Center for Cancer Research, National Cancer Institute, Frederick, MD 21702-1201, United States; Production Host Engineering Team, VTT Technical Research Centre of Finland Ltd, Maarintie 3, Espoo 02150, Finland; Department of Organic Chemistry, Institute of Chemistry, University of Campinas (UNICAMP), Rua Monteiro Lobato 270, Campinas, São Paulo 13.083-862, Brazil; Computational Biology Lab, National Agri-Food and Biomanufacturing Institute (NABI), Sector 81, S.A.S. Nagar, Mohali, Punjab 140306, India; Department of Paleobiotechnology, Leibniz Institute for Natural Product Research and Infection Biology Hans Knöll Institute, Beutenbergstraße 11A, Jena 07745, Germany; Institute for Organic Chemistry and BMWZ, Leibniz Universität Hannover, Schneiderberg 38, Hannover 30167, Germany; Translational Genome Mining for Natural Products, Interfaculty Institute of Microbiology and Infection Medicine Tübingen (IMIT), Interfaculty Institute for Biomedical Informatics (IBMI), University of Tübingen, Auf der Morgenstelle 24, Tübingen 72076, Germany; Translational Genome Mining for Natural Products, Interfaculty Institute of Microbiology and Infection Medicine Tübingen (IMIT), Interfaculty Institute for Biomedical Informatics (IBMI), University of Tübingen, Auf der Morgenstelle 24, Tübingen 72076, Germany; Institute of Applied Sciences, Korea Advanced Institute of Science and Technology (KAIST), Daejeon 34141, Republic of Korea; Bioinformatics Group, Wageningen University & Research, Droevendaalsesteeg 1, Wageningen 6708PB, The Netherlands; Computational Biology Lab, National Agri-Food and Biomanufacturing Institute (NABI), Sector 81, S.A.S. Nagar, Mohali, Punjab 140306, India; Regional Centre for Biotechnology, NCR Biotech Science Cluster, 3rd Milestone, Faridabad–Gurugram Expressway, Faridabad, Haryana (NCR Delhi) 121001, India; Computational Biology Lab, National Agri-Food and Biomanufacturing Institute (NABI), Sector 81, S.A.S. Nagar, Mohali, Punjab 140306, India; Bioinformatics Group, Wageningen University & Research, Droevendaalsesteeg 1, Wageningen 6708PB, The Netherlands; Bioinformatics Group, Wageningen University & Research, Droevendaalsesteeg 1, Wageningen 6708PB, The Netherlands; Department of Biotechnology and Nanomedicine, SINTEF Industry, P.O. Box 4760 Torgard, Trondheim N-7465, Norway; Computational Biology Lab, National Agri-Food and Biomanufacturing Institute (NABI), Sector 81, S.A.S. Nagar, Mohali, Punjab 140306, India; Computational Biology Lab, National Agri-Food and Biomanufacturing Institute (NABI), Sector 81, S.A.S. Nagar, Mohali, Punjab 140306, India; Regional Centre for Biotechnology, NCR Biotech Science Cluster, 3rd Milestone, Faridabad–Gurugram Expressway, Faridabad, Haryana (NCR Delhi) 121001, India; Computational Biology Lab, National Agri-Food and Biomanufacturing Institute (NABI), Sector 81, S.A.S. Nagar, Mohali, Punjab 140306, India; Department of Chemistry, University of Zurich, Winterthurerstrasse 190, Zurich 8057, Switzerland; Department of Chemical and Pharmaceutical Biology, University of Groningen, Antonius Deusinglaan 1, Groningen 9713AV, The Netherlands; Department of Pharmaceutical Sciences, University of Basel, Klingelbergstrasse 50, Basel 4056, Switzerland; The Novo Nordisk Foundation Center for Biosustainability, Technical University of Denmark, Building 220, Søltofts Plads, Kongens Lyngby 2800, Denmark; Bioinformatics Group, Wageningen University & Research, Droevendaalsesteeg 1, Wageningen 6708PB, The Netherlands; Department of Pharmaceutical Sciences, University of Basel, Klingelbergstrasse 50, Basel 4056, Switzerland; Institute of Plant and Food Science, Department of Biology, School of Life Sciences, Southern University of Science and Technology, 1088 Xueyuan Avenue, Shenzhen 518055, P.R. China; Translational Genome Mining for Natural Products, Interfaculty Institute of Microbiology and Infection Medicine Tübingen (IMIT), Interfaculty Institute for Biomedical Informatics (IBMI), University of Tübingen, Auf der Morgenstelle 24, Tübingen 72076, Germany; German Center for Infection Research (DZIF), Partner Site Tübingen, 72076 Tübingen, Germany; The Novo Nordisk Foundation Center for Biosustainability, Technical University of Denmark, Building 220, Søltofts Plads, Kongens Lyngby 2800, Denmark; The Novo Nordisk Foundation Center for Biosustainability, Technical University of Denmark, Building 220, Søltofts Plads, Kongens Lyngby 2800, Denmark; Bioinformatics Group, Wageningen University & Research, Droevendaalsesteeg 1, Wageningen 6708PB, The Netherlands; Department of Biochemistry, University of Johannesburg, C2 Lab Building 224, Kingsway Campus, Cnr University & Kingsway Road, Auckland Park, Johannesburg 2006, South Africa; Bioinformatics Group, Wageningen University & Research, Droevendaalsesteeg 1, Wageningen 6708PB, The Netherlands; Bioinformatics Group, Wageningen University & Research, Droevendaalsesteeg 1, Wageningen 6708PB, The Netherlands

## Abstract

Secondary or specialized metabolites show extraordinary structural diversity and potent biological activities relevant for clinical and industrial applications. The biosynthesis of these metabolites usually starts with the assembly of a core ‘scaffold’, which is subsequently modified by tailoring enzymes to define the molecule’s final structure and, in turn, its biological activity profile. Knowledge about reaction and substrate specificity of tailoring enzymes is essential for understanding and computationally predicting metabolite biosynthesis, but this information is usually scattered in the literature. Here, we present MITE, the Minimum Information about a Tailoring Enzyme database. MITE employs a comprehensive set of parameters to annotate tailoring enzymes, defining substrate and reaction specificity by the expressive reaction SMARTS (Simplified Molecular Input Line Entry System Arbitrary Target Specification) chemical pattern language. Both human and machine readable, MITE can be used as a knowledge base, for *in silico* biosynthesis, or to train machine-learning applications, and tightly integrates with existing resources. Designed as a community-driven and open resource, MITE employs a rolling release model of data curation and expert review. MITE is freely accessible at https://mite.bioinformatics.nl/.

## Graphical Abstract

**Graphical Abstract:**
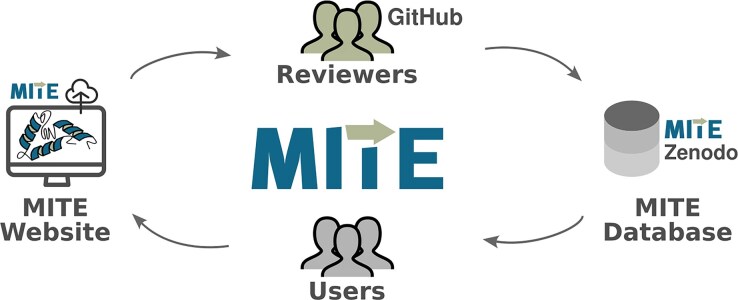

## Introduction

Many organisms can produce small molecules with intricate chemical structures and potent biological activities, known as specialized or secondary metabolites (SMs). Besides their widespread therapeutic and industrial application [1], SMs have high environmental relevance and are generally considered to grant an evolutionary advantage to the producing organism [2]. In microorganisms, their biosynthesis is often genetically organized in biosynthetic gene clusters (BGCs), sets of physically clustered genes encoding enzymes, transporters, and regulators that are collectively responsible for the controlled production of SMs [3]. Following the definitions of Walsh [4], the enzymes directly involved in the biosynthesis can be generally separated into gatekeeper and tailoring enzymes. Firstly, gatekeeper enzymes (also known as core enzymes) redirect primary metabolism building blocks into SM biosynthesis, forming the scaffold of the molecules [4]. Next, tailoring enzymes modify the nascent metabolite, eventually leading to its mature structure. These chemical modifications are often essential for bioactivity and target affinity [5, 6] and may have additional effects, such as enhanced chemical stability or solubility [4]. Therefore, detailed information about the reaction specificity and substrate promiscuity of tailoring enzymes, which can vary considerably even for closely related enzymes, is essential to understand and direct SM biosynthesis. Furthermore, it constitutes the foundation of algorithms for the prediction of chemical structures and biological activities directly from genomic data [7, 8], the investigation of quantitative structure–activity relationships, and the engineering of biosynthetic pathways [9].

However, details on tailoring enzymes, their reactions, and substrate specificities are usually deposited in narrative scientific articles, hampering computational access. A few databases exist that contain information on tailoring enzymes, but they are limited in their level of detail. While the Minimum Information about a Biosynthetic Gene Cluster (MIBiG) database provides information on BGCs including tailoring enzymes [10], their functional characterization is more focused on the scaffold-forming enzymes (e.g. non-ribosomal peptide synthetases), and tailoring enzyme descriptions are limited to generic terms. Some of the reactions contained in the Rhea database are associated with tailoring enzymes [11], but Rhea’s focus on stoichiometrically balanced and generalized reactions using a defined set of reactants is not always applicable to partially characterized and highly specific SM pathways, in which the precise order of reactions and therefore the precise substrate and product per reaction is often difficult to resolve. Databases such as Expasy ENZYME [12], KEGG [13], or BRENDA [14] also characterize tailoring enzymes, but are limited by the rigidity of the used EC (Enzyme Commission) number reaction classification system [15]. Hence, there is a need for a resource that provides flexible, comprehensive descriptions of tailoring enzymes and their substrate and reaction specificities linked to their genomic context, without relying on predefined reactants.

Here, we present the Minimum Information about a Tailoring Enzyme (MITE) database, dedicated to the characterization of SM-acting tailoring enzymes. MITE summarizes experimental data on the reactions and substrate specificities of these tailoring enzymes in both human- and machine-readable forms, using the reaction SMARTS notation (https://www.daylight.com/dayhtml/doc/theory/theory.smarts.html). This well-established chemical transformation language allows encoding enzymatic substrate recognition and modification as atom-bond patterns, allowing for flexible and concise description of substrate and reaction specificities. MITE is permissive towards unknown intermediates and ambiguous reaction order, and provides genomic context by specifying co-acting enzymes. MITE tightly integrates with established resources such as UniProt [16], NCBI GenBank [17], MIBiG [10], and Rhea [11], and can be used as a knowledge base, as a reference database for enzyme annotations (already in use by the genome mining tool antiSMASH [18]), and as a dataset for machine learning. With applications in pathway annotation, phylogenetic analyses, synthetic biology, metabolic engineering, and drug discovery, we expect MITE to be a highly beneficial database for both computational scientists and experimentalists alike. MITE is freely available at https://mite.bioinformatics.nl/.

## MITE database outline and infrastructure

### Data collection and database content

**Figure 1.**
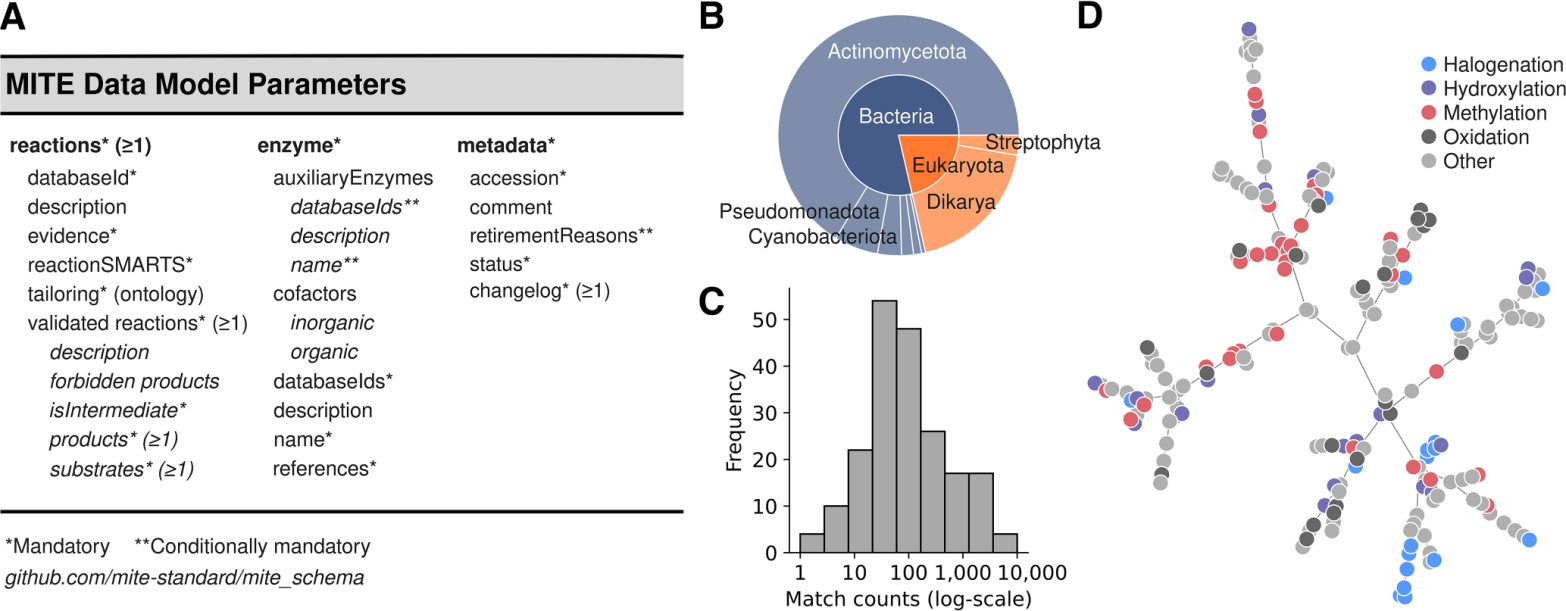
MITE data standard and database content. Summary of the MITE data model in panel (**A**), indicating mandatory and optional parameters. Panel (**B**) shows a sunburst plot of MITE entries, for which taxonomic information could be retrieved from NCBI Entrez (*n* = 187), with kingdom (inner circle) and phylum (outer circle) information. Panel (**C**) shows a histogram of distribution of BLASTp matches of MITE entries (*n* = 202) against the NCBI non-redundant protein database (at 70% sequence similarity cut-off, maximum 5000 matches per entry). Panel (**D**) shows a TMAP plot, with nodes representing DRFP-encoded example reactions from MITE entries (*n*= 202), annotated with tailoring function labels.

As an expert-curated database, MITE exclusively contains information on tailoring enzymes sourced from primary literature. Enzymes are within the scope of MITE if they directly partake in SM biosynthesis but are not ‘core’ or ‘gatekeeper’ enzymes (for a detailed discussion, see Walsh [4]). Therefore, transporter or resistance-conferring enzymes are excluded, as are multi-domain synthases and other classical scaffold-forming enzymes. Inspired by the MIBiG database [10], the MITE data model specifies a compact set of mandatory and optional parameters (Fig. 1A). Briefly, each MITE entry represents a single tailoring enzyme encoded by a single gene (one entry per protein isoform, if applicable) and is assigned a permanent identifier for consistent referencing. Protein sequences are referenced from either UniProt [16] or NCBI GenBank [17], and optional database cross-links to MIBiG [10] or Wikidata [19] can be specified. Additional ‘auxiliary’ enzymes may be referenced if required for the proper function of the described tailoring enzyme (e.g. macrolactam formation by McjC in the biosynthetic pathway of microcin J25 requires the presence of protease McjB [5]). Each MITE entry must also contain at least one reaction description in the form of a reaction SMARTS as an abstract representation of the substrate specificity and the reaction of the enzyme. This atom-bond chemical transformation pattern may represent discrete molecules or chemical substructures (e.g. the chlorination of an indole functional group or a tryptophan-containing peptide; see Supplementary Fig. S1 and Supplementary Table S1). Since reaction SMARTS may also represent non-viable substructures and contain wildcard characters or Boolean logic, they must be accompanied by an example reaction of discrete reactants in SMILES format, used to validate the reaction SMARTS. This reaction example is flexible and may also represent an R-group attached to a (partially known or unknown) core molecule (the latter represented as an asterisk), to be permissive to pathways where the exact order of reactions is not fully clear (e.g. Supplementary Fig. S1, see reaction example 1c). Reactions may be cross-referenced with a Rhea [11] reaction identifier or an EC number, and automation is in place to check for missing identifiers. Each entry must include at least one digital object identifier to primary literature (including preprints).

In its current version (1.16 [20]), the MITE database contains 202 ‘active’ entries, amounting to a total of 2639 data points (defined as key-value pairs, excluding metadata specified in Fig. 1A), including 283 reaction SMARTS representations and 291 example reactions. Entries show a wide taxonomic distribution (Fig. 1B), with most enzymes being associated with phyla Actinomycetota (66%) and Dikarya (19%). While 89% of MITE entries can be cross-referenced with a BGC from the MIBiG database, only 15% of enzymes are also covered by Rhea, and 7% could not be cross-referenced to either of the two databases (Supplementary Fig. S2). To functionally characterize the MITE dataset, we first investigated the sequence diversity of the covered enzymes. A comparison against the NCBI non-redundant protein database at a 70% sequence similarity cut-off (analogous to antiSMASH’s cut-off for comparison against the MITE database [18]) matched 77 461 protein sequences (Fig. 1C; see Supplementary Table S2). A sequence similarity network (SSN) of MITE entries (Supplementary Fig. S3) showed the formation of a few subnetworks, with the largest annotated as cytochrome P450, radical *S*-adenosyl-l-methionine enzymes, and flavin-dependent halogenases, although 46% of enzymes were ‘orphans’ with no next neighbours. When the SSN was annotated with tailoring function terms, sequence-related enzymes typically shared the same functional label, except for cytochrome P450 enzymes, which are known for their reaction diversity [21]. To get further insight into the represented biosynthetic reaction space, we sampled an example reaction from each MITE entry, generated bit fingerprints using the differential reaction fingerprint DRFP [22], clustered fingerprints using a *k*-nearest neighbour algorithm, visualized them using TMAP [23], and annotated them with tailoring reaction terms (Supplementary Fig. S4). The resulting graph (Fig. 1D) represents the diversity of newly generated substructures [i.e. the substructure difference between reactant(s) and product(s)], with next neighbours showing reaction similarity. While some aggregations of nodes indicating reaction similarity could be observed (e.g. halogenations), their clustering was not as distinct as in the SSN, with a wider dispersion of nodes indicating a diverse reaction space. Manual inspection of halogenation-labelled entries (Supplementary Fig. S5) confirmed reaction similarity of next neighbours exemplified by tryptophan halogenases, even though their substrate specificity could still vary considerably (e.g. MibH requires a full peptidic substrate, while PyrH acts on free tryptophan, even though they are both tryptophan 5-halogenases and thus next neighbours in the TMAP plot).

### Web interface and infrastructure

The MITE database is available as a collection of JavaScript Object Notation (JSON) files following a bespoke JSON Schema (https://json-schema.org/) data model. The JSON files are stored on Zenodo (https://zenodo.org/) [20], serving as the single source of truth. To facilitate interaction with the data, we developed the MITE web server implemented as a Flask (https://flask.palletsprojects.com/) web application, running within a Docker container (https://www.docker.com/). On startup, the web server populates the displayed data by downloading the most recent release of the MITE dataset from its Zenodo record. A non-persistent PostgreSQL (https://www.postgresql.org/) database is implemented to allow for complex search queries. The front end is implemented using Bootstrap (https://getbootstrap.com/), and the MITE website can be also viewed using mobile or tablet devices.

**Figure 2.**
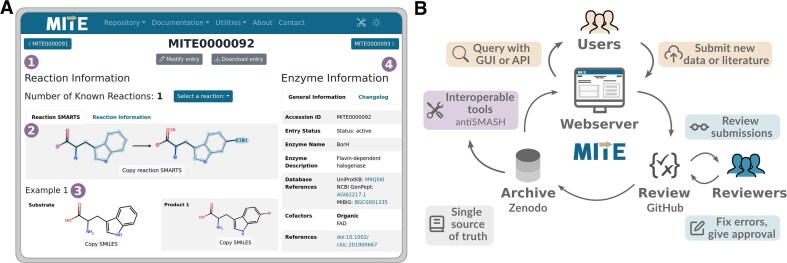
MITE web interface and infrastructure. Panel (**A**) shows an example entry page with (i) navigation bar, (ii) abstract substrate and reaction specificity visualized from reaction SMARTS, (iii) concrete example of substrate–product pair, and (iv) general enzyme information including changelog. Panel (**B**) shows a schema of MITE’s infrastructure, with circular data flow. Users can query data using the graphical user interface web server or API. Users can also create new or modify existing entries, which are automatically validated and undergo expert review. Approved entries are archived as releases on Zenodo, which acts as the single source of truth. From Zenodo, data are retrieved by the MITE web server or interoperable tools.

Central to the web server are the entry pages, which display the tailoring enzyme information in a dashboard-like format (Fig. 2A). The overview page allows for browsing entries and a range of search operations discussed below. All data can be downloaded as flat (text) files, and an application programming interface (API) following OpenAPI specifications (https://swagger.io/) is available for computational interactions. Besides data display and querying, the web server allows for the submission of new entries or the modification of existing ones, using integrated infrastructure (Fig. 2B). Online documentation and (video) tutorials are available to facilitate adoption by the community. Each contribution undergoes extensive automated validation (e.g. verifying the reaction SMARTS with the example reaction) and creates a new pull request on the GitHub page managing the MITE dataset, where it is reviewed by one of the expert reviewers. All contributions are automatically released to the public domain under the Creative Commons ‘No Rights Reserved’ license (https://creativecommons.org/public-domain/cc0/), which encourages and facilitates its reuse in other resources. The MITE database is updated regularly and new releases are automatically archived on the Zenodo data repository. From there, the MITE web server and interoperable tools retrieve the MITE entries in JSON format. The MITE database and accompanying infrastructure are governed by the MITE Standard GitHub organization (https://github.com/mite-standard), which also contains a forum for discussions and news items (e.g. web server downtime schedule). To ensure sustainability of the MITE database and minimize maintenance burden, social and technical workflows are in place, following O3 guidelines [24]. As a community-driven project, MITE welcomes participation in the form of data contributions, review, and governance participation. All contributors who have made a significant contribution [∼6 h of time investment, as specified in the MITE governance documentation (https://github.com/mite-standard/.github/blob/main/GOVERNANCE.md)] qualify for co-authorship.

### Applications of the MITE database

The MITE web server comes with a variety of search operations to query and subset data. Simple searches can be performed by querying the interactive overview table, while more complex queries can be constructed with the query builder, using Boolean logic. Queries can also be combined with substructure and reaction search using SMILES, SMARTS, or reaction SMARTS, and BLASTp searches for protein sequence similarity. For instance, a user could query entries annotated with ‘Methylation’ as tailoring reaction term and restrict the search to peptidic substrates using the SMARTS expression ‘[C](=[O]-[N-C-C](=[O]’ (Supplementary Fig. S6). As of 12 August 2025, using MITE data version 1.16 [20], this results in seven entries, which can be then browsed, further subsetted, or downloaded in tabular format for downstream analysis.

**Figure 3.**
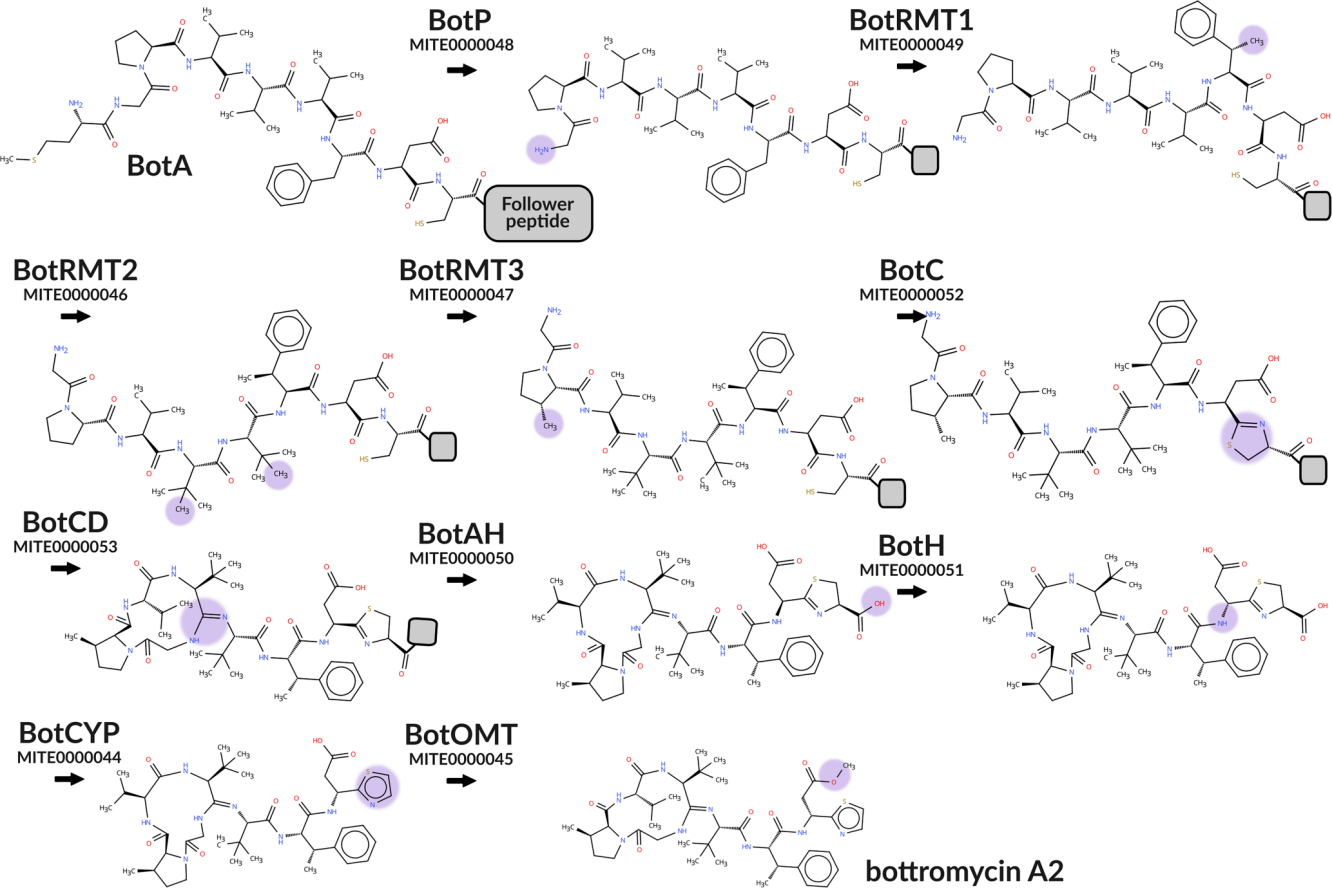
*In silico* biosynthesis of bottromycin A2. Enzymes involved in bottromycin A2 biosynthesis (MIBiG cluster BGC0000469) are represented by MITE entries. Starting from the precursor peptide substrate BotA, reaction SMARTS are applied consecutively, each including one or more tailoring reactions performed by the respective enzyme. Purple circles indicate the area of modification following the previous enzymatic biotransformation (e.g. epimerization by BotH). Eventually, this biosynthetic cascade results in the mature, literature-reported bottromycin A2 metabolite.

Another use case for the MITE data repository is for *in silico* simulation of SM biosynthesis. Since each MITE entry has one or more associated reaction SMARTS, these can be rapidly applied to substrate SMILES as ‘reaction rules’ with ‘built-in’ substrate recognition, acting as an abstraction of the enzymatic reaction. Combining multiple MITE entries thus allows to approximate biosynthetic pathways. As a proof of concept, we investigated the biosynthetic pathway of bottromycin A2, a thoroughly experimentally characterized metabolite modified by no fewer than 10 tailoring enzymes [25]. Starting from the peptidic precursor BotA (MIBiG cluster BGC0000469), biosynthesis can be simulated stepwise in an automated fashion, using MITE reaction SMARTS retrieved via the API (Fig. 3). As expected, the resulting final product matched the structure of the literature-reported bottromycin A2 [25]. To facilitate such explorative *in silico* biosynthesis, we provide a reaction planner on the MITE website (Supplementary Fig. S7).

### Discussion and future directions

The MITE database significantly advances SM research by providing a dedicated resource for the detailed characterization of tailoring enzymes. Representing reaction and substrate specificity in the reaction SMARTS format allows for compact descriptions of both discrete compounds as well as atom-bond patterns, exceeding the capabilities of traditional reactant-based representations of biological reactions. Chemical transformations can be expressed abstractly, accommodating arbitrary levels of substrate promiscuity and specificity. This includes the description of partial or full structures, the use of wildcard characters for atoms and bonds, and Boolean logic. The resulting expressions are easily searchable, compatible with a variety of cheminformatics and synthetic biology tools, and suited for machine-learning-based applications. Reaction SMARTS can be created and modified by various chemistry drawing programs [e.g. Ketcher (https://lifescience.opensource.epam.com/ketcher/), Marvin (https://chemaxon.com/marvin)] and invite gradual refinement over time, allowing to incorporate new knowledge on tailoring enzyme reaction specificity as it becomes available. MITE is designed following FAIR (findable, accessible, interoperable, reusable) data principles and facilitates access to machine-readable information on tailoring enzymes substrate promiscuity, previously only available through laborious manual search of scientific articles. MITE emphasized accessibility and interoperability by its use of standardized data formats and data exchange protocols, and is registered on Bioregistry [26] and FAIRsharing [27] platforms. MITE is already used as a reference database by version 8 of the popular genome mining tool antiSMASH [18].

Among existing databases, MITE shows the greatest similarity with the Rhea database. This resource also annotates tailoring enzymes, but it is mainly a biological reaction database, focusing on the representation of standardized, balanced ‘master’ reactions with clearly defined substrates and products intended for pathway mapping and metabolic modelling. In contrast, MITE is an enzyme database, allowing higher flexibility in representing catalytic functions. MITE is permissive to partially resolved structures without ChEBI identifiers [28], unknown co-factors and intermediates, and unclear reaction order characteristic of poorly investigated SM biosynthetic pathways. The enzyme coverage of the MITE database is clearly distinct from Rhea, and only 15% of enzymes characterized in MITE can be annotated with a Rhea entry. Despite the different scope of the resources, we believe that MITE and Rhea are actually highly complementary: for well-characterized tailoring enzymes, MITE can reference master reactions from Rhea, while MITE can provide chemical information on enzymes that are currently difficult to annotate by the standards of Rhea. MITE entries may be also suitable to serve as a resource for new Rhea entries, and annotation efforts from both resources are planned to reinforce each other, creating a virtuous circle of data enhancement. MITE also integrates with other resources, especially the BGC repository MIBiG, which uses MITE as a resource to annotate tailoring enzyme-encoding genes. Additional cross-links can be established by providing an EC number (e.g. https://bioregistry.io/mite:MITE0000029), or linking to a Wikidata enzyme entry, and automation during data submission is in place to support manual data curation with linking to external resources.

The MITE database also comes with some limitations, including the type of reaction SMARTS it accepts. While the Daylight company (https://www.daylight.com/) remains the central authority in defining reaction SMARTS, there is a proliferation of sometimes conflicting variants or ‘flavors’ employed by various tools, including proprietary extensions such as Chemaxon’s CXSMARTS. To provide a common ground compatible with a majority of downstream applications without potential licensing restrictions, MITE only accepts generic reaction SMARTS, and ‘standardizes’ them using the cheminformatics tool RDKit (https://www.rdkit.org). This validation takes place during community submission, and only reaction SMARTS passing this initial step are accepted, ensuring an interoperable and high-quality dataset.

Moving forward, we intend to further develop the coverage of MITE by participating in the upcoming MIBiG 5.0 hackathons and further encouraging community participation. Additionally, we will work on adding BGCs of the 7% of MITE entries that currently do not have an entry in MIBiG, further improving both resources. We will continue periodic cross-referencing with MIBiG, introduce automation that synchronizes with the Wikidata knowledge graph, and explore semiautomated data exchange with the Rhea database. We intend to continue developing the data submission workflow and extend the capability of the MITE’s API. We will also proceed exploring ways to make the MITE database compatible and accessible to machine-learning tools as training and/or test dataset.

In conclusion, the MITE database is a novel resource to characterize SM-acting tailoring enzymes. Following publication, MITE is expected to rapidly expand in coverage through its community-driven curation and rolling release system and is designed to be a sustainable, open, and interoperable source of knowledge. The researchers represented by the present publication commit to submitting MITE-compliant data when publishing experimental results on tailoring enzymes, and we encourage the broader research community to join the initiative. With applications as a knowledge base, parts catalog for synthetic biology, and a resource for phylogenetic analyses, genome mining, and pathway annotation, we expect MITE to be a highly beneficial database to further enhance knowledge on the enzymology and chemistry connected to SMs. The MITE database is available at https://mite.bioinformatics.nl/.

## Supplementary Material

gkaf969_Supplemental_Files

## Data Availability

The MITE database is released to the public domain under the Creative Commons ‘No Rights Reserved’ license (https://creativecommons.org/public-domain/cc0/) and available on https://mite.bioinformatics.nl/ and Zenodo [20]. The source code used for the web interface [29], the data model [30], and data validation [31] is available at https://github.com/mite-standard, licensed under the MIT License (https://opensource.org/license/mit). Source code for the generation of figures for this manuscript is available at https://github.com/mite-standard/mite_ms and also licensed under the MIT License.
