# Supplementary material for: MITE: the Minimum Information about a Tailoring Enzyme database for capturing specialized metabolite biosynthesis
Source: Nucleic Acids Res. 2025 Sep 26;54(D1):D635–42. doi: 10.1093/nar/gkaf969 (PMC12807718; doi:10.1093/nar/gkaf969)
Supplement: gkaf969_Supplemental_Files [file gkaf969_supplemental_files.zip › MITE_MS_07-25-SI-revision-v1.docx]

# **MITE: the Minimum Information about a Tailoring Enzyme database for capturing specialized metabolite biosynthesis - Supplementary Information**

Adriano Rutz^1^, Daniel Probst^2^, César Aguilar^3^, Daniel Y. Akiyama^4^, Fabrizio Alberti^5^, Hannah E. Augustijn^2,6^, Nicole E. Avalon^7,8^, Christine Beemelmanns^9,10^, Hellen Bertoletti Barbieri^4^, Friederike Biermann^2,11,12^, Alan J. Bridge^13^, Esteban Charria Girón^2,14^, Russell Cox^15^, Max Crüsemann^16,17^, Paul M. D'Agostino^9^, Marc Feuermann^13^, Jennifer Gerke^18^, Karina Gutiérrez García^19,20^, Jonathan E. Holme^21^, Ji-Yeon Hwang^22^, Riccardo Iacovelli^23^, Júlio César Jeronimo Barbosa^4^, Navneet Kaur^24^, Martin Klapper^25^, Anna M. Köhler^15^, Aleksandra Korenskaia^26^, Noel Kubach^26^, Byung T. Lee^27^, Catarina Loureiro^2^, Shrikant Mantri^24,28^, Simran Narula^24^, David Meijer^2^, Jorge C. Navarro-Muñoz^2^, Giang-Son Nguyen^21^, Sunaina Paliyal^24^, Mohit Panghal^24,28^, Latika Rao^24^, Simon Sieber^29^, Nika Sokolova^30^, Sven T. Sowa^31^, Judit Szenei^32^, Barbara R. Terlouw^2^, Heiner G. Weddeling^31^, Jingwei Yu^33^, Nadine Ziemert^26,34^, Tilmann Weber^32^, Kai Blin^32^, Justin J.J. van der Hooft^2,35^, Marnix H. Medema^2^*, Mitja M. Zdouc^2^*

^1^Institute for Molecular Systems Biology, ETH Zürich, Otto-Stern-Weg 3, 8093 Zürich, Switzerland

^2^Bioinformatics Group, Wageningen University & Research, Droevendaalsesteeg 1, 6708 PB Wageningen, the Netherlands

^3^Industrial Genomics Laboratory, Centro de Biotecnología FEMSA, Escuela de Ingeniería y Ciencias, Tecnológico de Monterrey, Av. Eugenio Garza Sada 2501 sur, Nuevo Leon, 64700, México

^4^Department of Organic Chemistry, Institute of Chemistry, University of Campinas (UNICAMP), Rua Monteiro Lobato 270, Campinas, São Paulo, 13.083-862, Brazil

^5^School of Life Sciences, University of Warwick, Gibbet Hill Road, Coventry, CV4 7AL, UK

^6^Institute of Biology, Leiden University, Sylviusweg 72, 2333BE Leiden, the Netherlands

^7^Department of Pharmaceutical Sciences, University of California, Irvine, 856 Health Sciences Road, Irvine, California, USA 92697

^8^Center for Marine Biotechnology and Biomedicine, Scripps Institution of Oceanography, University of California San Diego, 9500 Gilman Drive, La Jolla, CA 92093-0212, USA

^9^Helmholtz Institute for Pharmaceutical Research Saarland (HIPS), Helmholtz Centre for Infection Research (HZI), Campus E8.1, 66123 Saarbrücken, Germany

^10^Saarland University, Campus E8.1, 66123 Saarbrücken, Germany

^11^Institute for Molecular Biosciences, Goethe University Frankfurt, Max-von-Laue-Str. 9, 60438 Frankfurt am Main, Germany

^12^LOEWE Center for Translational Biodiversity Genomics (TBG), Senckenberganlage 25, 60325 Frankfurt am Main, Germany

^13^SIB Swiss Institute of Bioinformatics, Centre Medical Universitaire, 1 rue Michel Servet, 1211 Geneva 4, Switzerland

^14^Department of Microbial Drugs, Helmholtz Centre for Infection Research (HZI), Inhoffenstr. 7, 38124 Braunschweig, Germany

^15^Institute for Organic Chemistry and BMWZ, Leibniz Universität Hannover, Schneiderberg 38, 30167 Hannover, Germany

^16^Institute of Pharmaceutical Biology, University of Bonn, Nussallee 6, 53115 Bonn, Germany

^17^Institute for Pharmaceutical Biology, Goethe University Frankfurt, Max-von-Laue-Str. 9, 60438 Frankfurt am Main, Germany

^18^Institute for Organic Chemistry, Leibniz Universität Hannover, Schneiderberg 38, 30167 Hannover, Germany

^19^Biosphere Sciences and Engineering Division, Carnegie Institution for Science, 3520 San Martin Dr, Baltimore, MD, 21218, USA

^20^Department of Ecology and Evolutionary Biology, University of Arizona, 1041 E. Lowell St., Tucson, AZ 85721, USA

^21^Department of Biotechnology and Nanomedicine, SINTEF Industry, P.O.Box 4760 Torgard, N-7465 Trondheim, Norway

^22^Molecular Targets Program, Center for Cancer Research, National Cancer Institute, Frederick, Maryland 21702-1201, USA

^23^Production Host Engineering Team, VTT Technical Research Centre of Finland Ltd, Maarintie 3, 02150 Espoo, Finland

^24^Computational Biology Lab, National Agri-Food and Biomanufacturing Institute (NABI), Sector 81, S.A.S. Nagar, Mohali, Punjab 140306, India

^25^Department of Paleobiotechnology, Leibniz Institute for Natural Product Research and Infection Biology Hans Knöll Institute, Beutenbergstr. 11a, 07745 Jena, Germany

^26^Translational Genome Mining for Natural Products, Interfaculty Institute of Microbiology and Infection Medicine Tübingen (IMIT), Interfaculty Institute for Biomedical Informatics (IBMI), University of Tübingen, Auf der Morgenstelle 24, 72076 Tübingen, Germany

^27^Institute of Applied Sciences, Korea Advanced Institute of Science and Technology (KAIST), Daejeon 34141, Republic of Korea

^28^Regional Centre for Biotechnology, NCR Biotech Science Cluster, 3rd Milestone, Faridabad-Gurugram Expressway, Faridabad - 121001 Haryana (NCR Delhi), India

^29^Department of Chemistry, University of Zurich,Winterthurerstrasse 190, 8057 Zurich, Switzerland

^30^Department of Chemical and Pharmaceutical Biology, University of Groningen, Antonius Deusinglaan 1, 9713AV Groningen, The Netherlands

^31^Department of Pharmaceutical Sciences, University of Basel, Klingelbergstrasse 50, 4056 Basel, Switzerland

^32^The Novo Nordisk Foundation Center for Biosustainability, Technical University of Denmark, Building 220, Søltofts Plads, 2800 Kongens Lyngby, Denmark

^33^Institute of Plant and Food Science, Department of Biology, School of Life Sciences, Southern University of Science and Technology, 1088 Xueyuan Avenue, Shenzhen 518055, P.R. China

^34^German Center for Infection Research (DZIF), Partner Site Tübingen, Germany

^35^Department of Biochemistry, University of Johannesburg, C2 Lab Building 224, Kingsway Campus, Cnr University & Kingsway Road, Auckland Park, Johannesburg 2006, South Africa

*Co-corresponding authors: Marnix H. Medema (marnix.medema@wur.nl), Mitja M. Zdouc (mitja.zdouc@wur.nl).


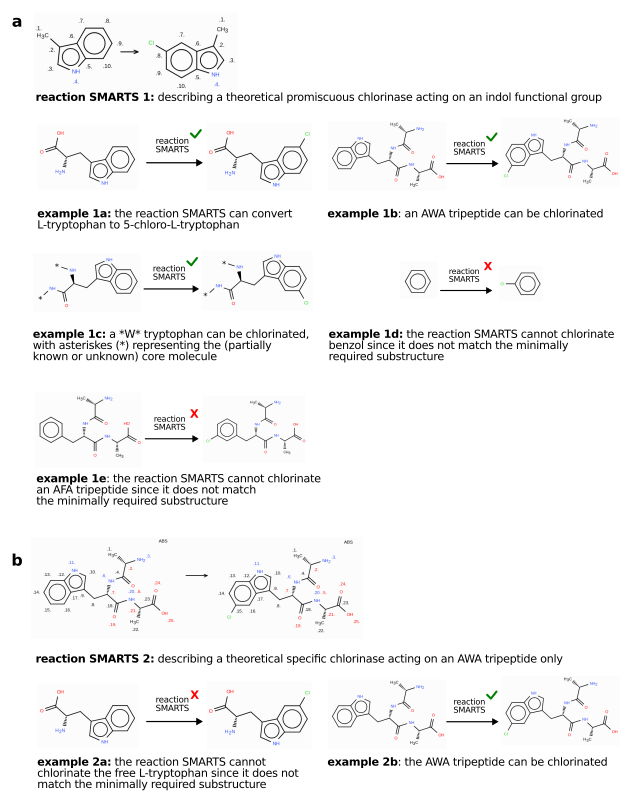


**Figure S1: Example reaction SMARTS and reaction examples.** Panel (a) shows reaction SMARTS 1, describing a theoretical promiscuous chlorinase that can chlorinate an indole functional group on any kind of substrate. Reaction SMARTS 1 can therefore modify substrates in example reaction 1a-1c, but not 1d and 1e, since they lack an indole functional group. Panel (b) shows reaction SMARTS 2, describing a theoretical chlorine with high specificity for an AWA tripeptide. This reaction SMARTS cannot modify the free L-tryptophan substrate of reaction example 2a, since it lacks the minimally required AWA structure. However, it can modify the substrate of example 2b.

**Table S1: Reaction SMARTS and example reaction SMILES.** Reaction SMARTS and SMILES used in the examples demonstrated in Fig. S1.

| **Identifier** | **Reaction SMARTS** | **Substrate SMILES** | **Product SMILES** |
| --- | --- | --- | --- |
| **reaction SMARTS 1** | [#6:1]-[#6:2]1:[#6:6]2:[#6:7]:[#6:8]:[#6:9]:[#6:10]:[#6:5]:2:[#7:4]:[#6:3]:1>>[#6:1]-[#6:2]1:[#6:6]2:[#6:7]:[#6:8](-[Cl]):[#6:9]:[#6:10]:[#6:5]:2:[#7:4]:[#6:3]:1 |  |  |
| **reaction 1a/2a** |  | N[C@@H](Cc1c[nH]c2ccccc12)C(=O)O | N[C@@H](Cc1c[nH]c2ccc(Cl)cc12)C(=O)O |
| **reaction 1b/2b** |  | C[C@H](N)C(=O)N[C@@H](Cc1c[nH]c2ccccc12)C(=O)N[C@@H](C)C(=O)O | C[C@H](N)C(=O)N[C@@H](Cc1c[nH]c2ccc(Cl)cc12)C(=O)N[C@@H](C)C(=O)O |
| **reaction 1c** |  | *NC(=O)[C@H](Cc1c[nH]c2ccccc12)N* | *NC(=O)[C@H](Cc1c[nH]c2ccc(Cl)cc12)N* |
| **reaction 1d** |  | c1ccccc1 | c1ccc(Cl)cc1 |
| **reaction 1e** |  | C[C@@H](C(N[C@H](C(N[C@H](C(O)=O)C)=O)Cc1ccccc1)=O)N | C[C@@H](C(N[C@H](C(N[C@H](C(O)=O)C)=O)Cc1cc(Cl)ccc1)=O)N |
| **Reaction SMARTS 2** | [#6:1]\[#6@@:2](-[#6:4](-[#7:6]\[#6@:7](-[#6:18](-[#7:20]-[#6@:21](-[#6:23](-[#8:25])=[#8:24])\[#6:22])=[#8:19])-[#6:8]-[#6:9]1:[#6:17]2:[#6:12](:[#6:13]:[#6:14]:[#6:15]:[#6:16]:2):[#7:11]:[#6:10]:1)=[#8:5])-[#7:3]>>[#6:1]\[#6@@:2](-[#6:4](-[#7:6]\[#6@:7](-[#6:18](-[#7:20]-[#6@:21](-[#6:23](-[#8:25])=[#8:24])\[#6:22])=[#8:19])-[#6:8]-[#6:9]1:[#6:17]2:[#6:12](:[#6:13]:[#6:14]:[#6:15](-[Cl]):[#6:16]:2):[#7:11]:[#6:10]:1)=[#8:5])-[#7:3] |  |  |

**
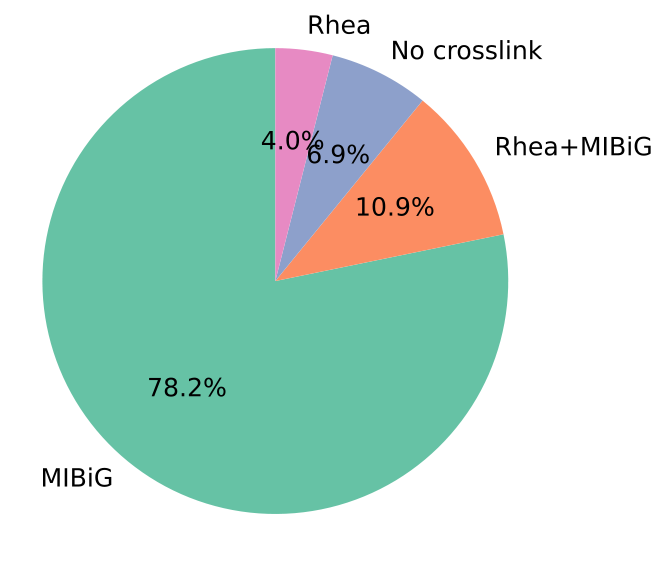
**

**Figure S2: Pie chart of selected database crosslinks.** Each MITE entry with the “status=active” flag (n=202) was searched for a Rhea ID or MIBiG ID database cross-reference. Entries with missing cross-references were validated by querying against the respective database (MIBiG: search for matching NCBI Genpept ID, Rhea: search for a matching UniProt ID). All computer code describing the generation of the pie-chart can be found at https://github.com/mite-standard/mite_ms. The pie-chart was exported as an scalable vector graphics (SVG) image and arranged using Inkscape (https://inkscape.org/).

**Table S2: Descriptive statistics for histogram in Fig. 1c.** For each MITE entry with the “status=active” flag (n=202), its associated protein sequence was queried against the NCBI non-redundant protein sequences database (version 31/01/2025) using NCBI API BLASTp (expect=1e-5, hitlist_size=5000). Only pairs with a sequence similarity of ≥70% (positives/alignment length * 100) were retained, counted as “ncbi_nr_matches” and plotted as histogram shown in Fig. 1c. All computer code describing the generation of the histogram can be found at <https://github.com/mite-standard/mite_ms>. The histogram was exported as an SVG image and arranged using Inkscape (<https://inkscape.org/>).

| **description** | **value** |
| --- | --- |
| MITE entries (only with status=active) | 202 |
| Mean nr of matches vs NCBI NR | 383.47 |
| Standard deviation | 828.49 |
| 1st quartile nr of matches | 28.0 |
| 2nd quartile nr of matches | 72.5 |
| 3rd quartile nr of matches | 221.75 |
| Maximum nr of matches | 4999 |
| Interquartile range | 193.75 |
| Lower bound | -262.625 |
| Upper bound | 512.375 |


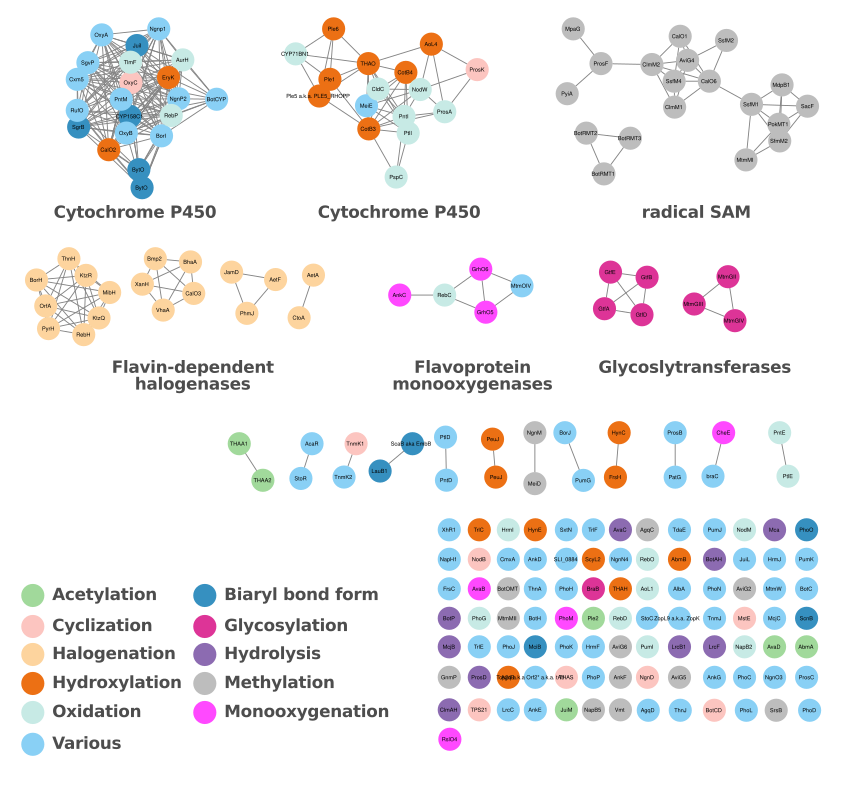


**Figure S3: Sequence similarity network of MITE entries.** EFI-EST [(1)](https://paperpile.com/c/ud9mVz/Q2jY) was used to generate a sequence similarity network of MITE entries with the “status=active” flag (n=202), with settings E-value 5 and alignment score 20. The resulting network was visualized using Cytoscape [(2)](https://paperpile.com/c/ud9mVz/P8ef) with the yFiles Organic Layout, annotated with tailoring reaction terms (see Figure S4). Terms with a frequency of <=5 were merged into “Various”, including the “Other” tailoring term. The network was exported as an SVG image and arranged using Inkscape (<https://inkscape.org/>).


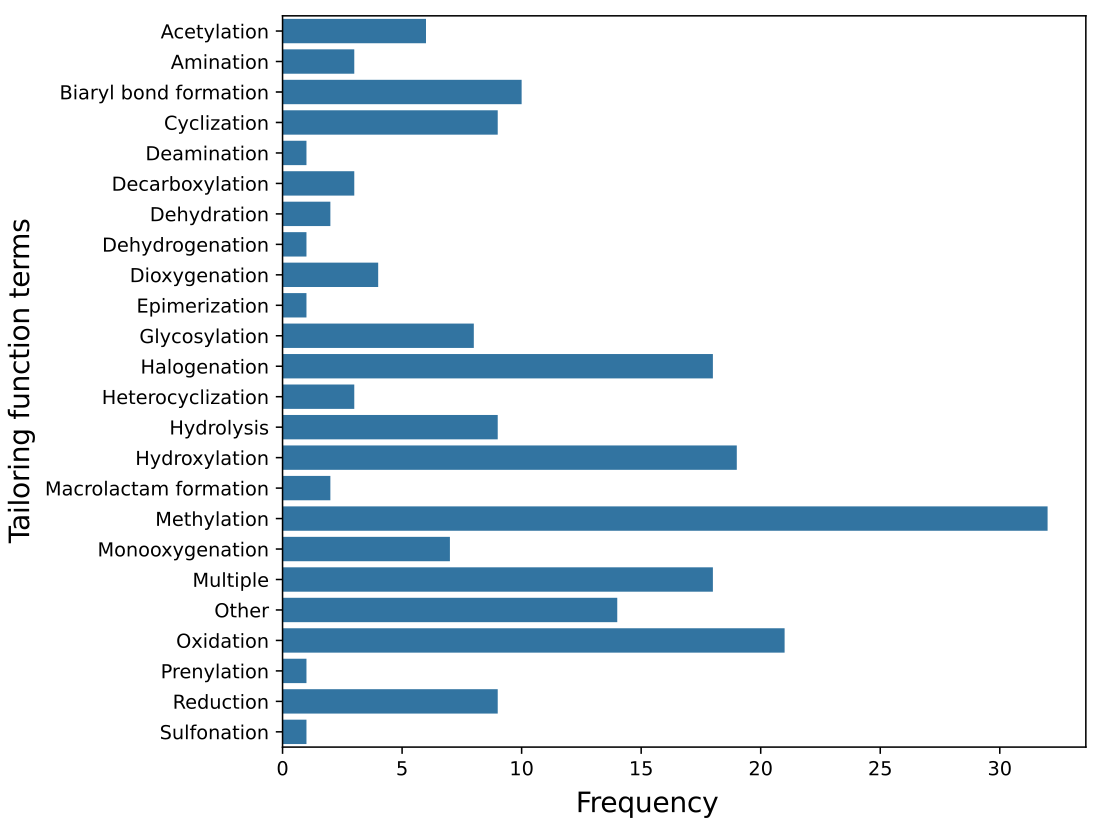


**Figure S4: Barplot of tailoring reaction terms.** Each MITE entry with the “status=active” flag (n=202) was searched for its tailoring reaction terms, which were summed and plotted as a barplot. MITE entries with more than one tailoring reaction term were summarized using the label “Multiple”. All computer code describing the generation of the barplot can be found at <https://github.com/mite-standard/mite_ms>. The barplot was exported as an SVG image and arranged using Inkscape (<https://inkscape.org/>).


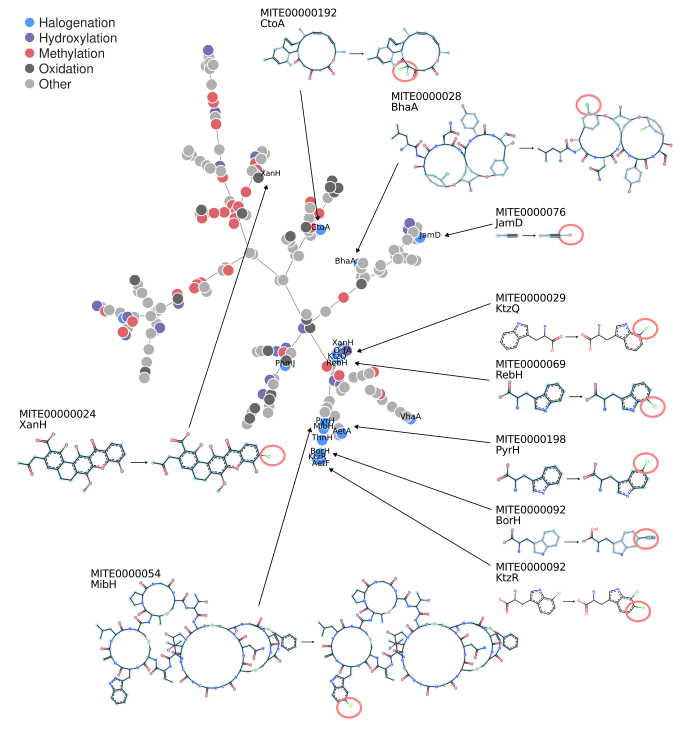


**Figure S5: Annotated TMAP plot from Fig. 1d.** TMAP plot, with nodes representing DRFP-encoded example reactions from MITE entries, annotated with tailoring reaction labels and enzyme names. Reaction SMARTS visualizations of selected entries are superimposed. While the formation of a few local concentrations of similar entries can be seen in the TMAP for halogenation reactions, the substrate- and reaction specificity shows variability. The network was visualized using Seaborn’s scatter plot functionality. The network including annotations was exported as SVG image and arranged using Inkscape (<https://inkscape.org/>). All computer code describing the generation of the TMAP plot can be found at <https://github.com/mite-standard/mite_ms>.


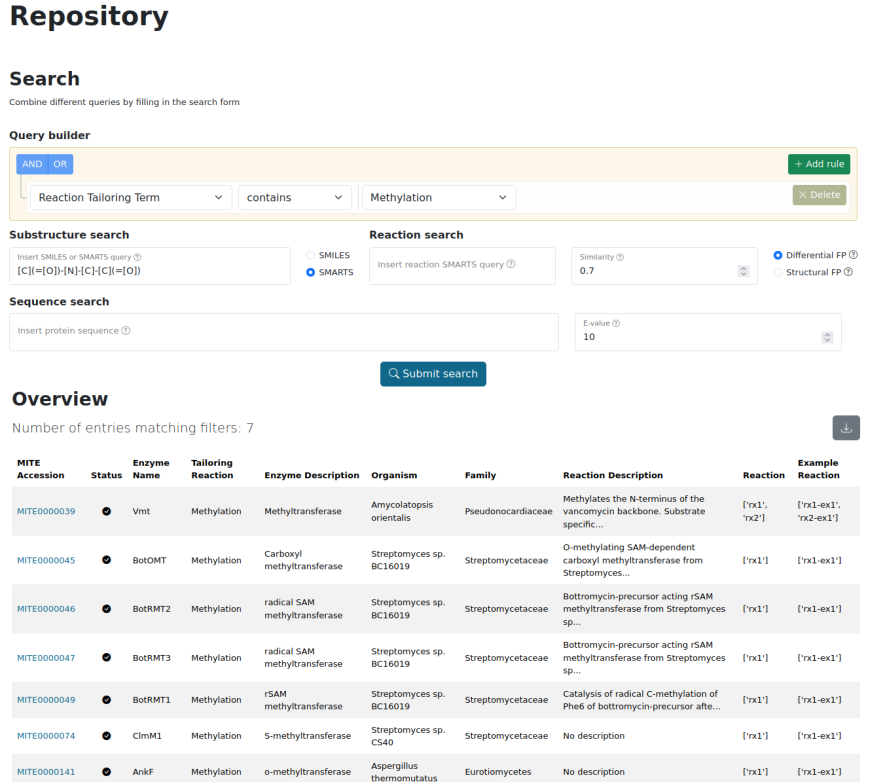


**Figure S6: Example query on MITE Overview page.** The MITE database can be queried with a combination of simple search, substructure search with SMILES and SMARTS, reaction search using reaction SMARTS, and protein sequence similarity. Filters can be combined, allowing for complex queries. In this example query, the reaction tailoring term “Methylation” was combined with a SMARTS expression for a peptide bond, resulting in seven entries matching the filter.

**
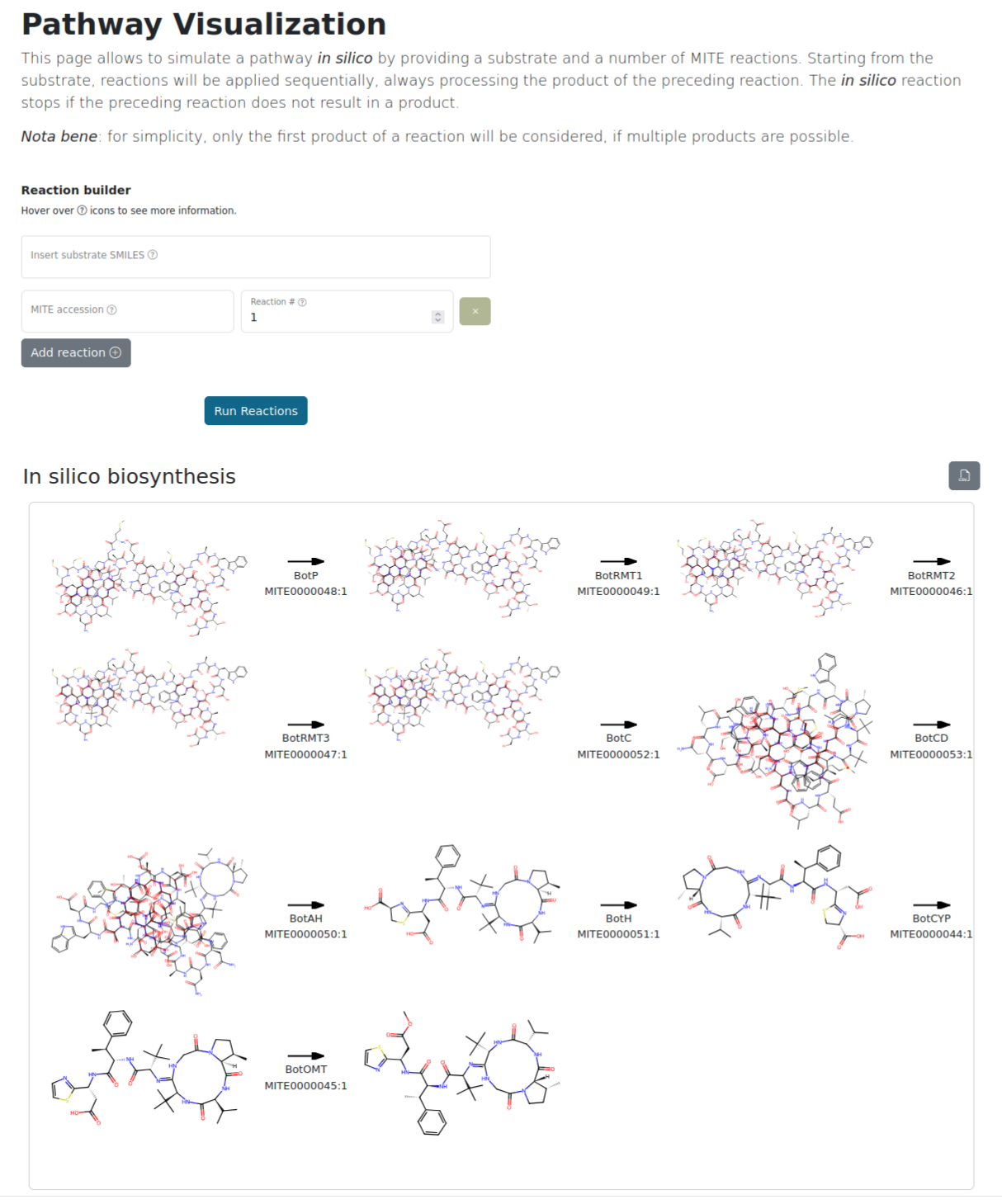
**

**Figure S7: Example *in silico* biosynthesis on MITE Pathway Visualization page.** The MITE webserver offers the possibility to visualize pathways and simulate biosynthesis *in silico*. In this example, the bottromycin A2 pathway was simulated, starting from the bottromycin precursor peptide BotA (MIBiG cluster [BGC0000469](https://mibig.secondarymetabolites.org/repository/BGC0000469.6/index.html#r1c1)). The resulting visualization can be used to prepare custom figures, and a report containing substrate and product SMILES in .scv-format can be downloaded for downstream processing.

**References**

[1. Zallot,R., Oberg,N. and Gerlt,J.A. (2019) The EFI Web Resource for Genomic Enzymology Tools: Leveraging Protein, Genome, and Metagenome Databases to Discover Novel Enzymes and Metabolic Pathways. *Biochemistry*,](http://paperpile.com/b/ud9mVz/Q2jY) [10.1021/acs.biochem.9b00735](http://dx.doi.org/10.1021/acs.biochem.9b00735)[.](http://paperpile.com/b/ud9mVz/Q2jY)

[2. Shannon,P., Markiel,A., Ozier,O., Baliga,N.S., Wang,J.T., Ramage,D., Amin,N., Schwikowski,B. and Ideker,T. (2003) Cytoscape: a software environment for integrated models of biomolecular interaction networks. *Genome Res*, **13**, 2498–2504.](http://paperpile.com/b/ud9mVz/P8ef)
